# Acceptability and feasibility of community-based provision of urine pregnancy tests to support linkages to reproductive health services in Western Kenya: a qualitative analysis

**DOI:** 10.1186/s12884-022-04869-8

**Published:** 2022-09-01

**Authors:** Mia Kibel, Julie Thorne, Caroline Kerich, Violet Naanyu, Faith Yego, Astrid Christoffersen-Deb, Caitlin Bernard

**Affiliations:** 1 Department of Obstetrics & Gynaecology, Temerty Faculty of Medicine, University of Toronto, Suite 1200, 123 Edward Street, Toronto, ON M5G 1E2 Canada; 2 Academic Model Providing Access to Healthcare (AMPATH), P.O Box 4606-30100, Eldoret, Kenya; 3 Department of Reproductive Health, School of Medicine, Moi University, P.O. Box 4606-30100, Eldoret, Kenya; 4 Department of Health Policy and Management, School of Public Health, College of Health Sciences, Moi University, P.O. Box 4606-30100, Nandi Road, Eldoret, Kenya; 5 Department of Sociology, Psychology and Anthropology, School of Arts and Social Sciences, Moi University, P.O.Box 3900-30100, Eldoret, Kenya; 6 Department of Obstetrics and Gynecology, Faculty of Medicine, University of British Columbia, Suite 930, 1125 Howe Street, Vancouver, BC V6Z 2K8 Canada; 7 Department of Obstetrics & Gynecology, Indiana University School of Medicine, University Hospital 2440, 550 University Boulevard, Indianapolis, IN 46202 USA

**Keywords:** Reproductive Health Services, Family Planning Services, Community Health Workers, Kenya

## Abstract

**Background:**

The majority of women living in rural Kenya access antenatal care (ANC) late in pregnancy, and approximately 20% have an unmet need for family planning (FP). This study aimed to determine whether training community health volunteers (CHVs) to deliver urine pregnancy testing (UPT), post-test counselling, and referral to care was an acceptable and feasible intervention to support timely initiation of ANC and uptake of FP.

**Methods:**

We applied community-based participatory methods to design and implement the pilot intervention between July 2018 and May 2019. We conducted qualitative content analysis of 12 pre-intervention focus group discussions (FGDs) with women, men, and CHVs, and of 4 post-intervention FGDs with CHVs, each with 7–9 participants per FGD group. Using a pragmatic approach, we conducted inductive line-by-line coding to generate themes and subthemes describing factors that positively or negatively contributed to the intervention’s acceptability and feasibility, in terms of participants’ views and the intervention aims.

**Results:**

We found that CHV-delivered point of care UPT, post-test counselling, and referral to care was an acceptable and feasible intervention to increase uptake of ANC, FP, and other reproductive healthcare services. Factors that contributed to acceptability were: (1) CHV-delivery made UPT more accessible; (2) UPT and counselling supported women and men to build knowledge and make informed choices, although not necessarily for women with unwanted pregnancies interested in abortion; (3) CHVs were generally trusted to provide counselling, and alternative counselling providers were available according to participant preference. A factor that enhanced the feasibility of CHV delivering UPT and counselling was CHV's access to appropriate supplies (e.g. carrying bags). However, factors that detracted from the feasibility of women actually accessing referral services after UPT and counselling included (1) downstream barriers like cost of travel, and (2) some male community members’ negative attitudes toward FP. Finally, improved financial, educational, and professional supports for CHVs would be needed to make the intervention acceptable and feasible in the long-term.

**Conclusion:**

Training CHVs in rural western Kenya to deliver UPT, post-test counselling, and referral to care was acceptable and feasible to men, women, and CHVs in this context, and may promote early initiation of ANC and uptake of FP. Additional qualitative work is needed to explore implementation challenges, including issues related to unwanted pregnancies and abortion, the financial burden of volunteerism on CHVs, and educational and professional supports for CHVs.

**Supplementary Information:**

The online version contains supplementary material available at 10.1186/s12884-022-04869-8.

## Background

The World Health Organization (WHO) identifies access to appropriate and timely antenatal care (ANC) and family planning (FP) services as cornerstones of primary care.[1] Access to ANC in the first four months of pregnancy is important for early identification of pregnancy risk factors, HIV testing and treatment, malaria prevention, and linkage to a health facility for delivery [1, 2]. Access to timely and appropriate FP services reduces maternal mortality by preventing unwanted high risk pregnancies and unsafe abortions [3]. Access to both ANC and FP have been identified as fundamental to individuals’ health and human rights [4].

In Kenya, a substantial proportion of women, particularly in rural areas, do not have access to appropriate and timely ANC and FP. In 2014, only 16% of reproductive age women in rural Kenya had their first ANC visit by 16 weeks of pregnancy, and 20% had an unmet need for FP [5]. Disparities in care are driven by multiple factors, including distance to health facilities, sociocultural factors limiting demand, and poverty [6]. Creative, cost-effective tools that address these barriers are urgently needed to increase access to and uptake of ANC and FP in rural areas.

The 2014–2030 Kenya Health Policy outlines a number of strategies to increase access to ANC and FP [7]. At the Community level, the policy aims to create appropriate demand for healthcare services and establish strong links between communities and primary care services. Community level efforts are organized around Community Health Units (CHU), which are groups of households organized by village or sub-location. Within a CHU, health initiatives are directed by a Community Health Officer and executed by Community Health Volunteers (CHVs), who are members of the local community usually selected at community meetings. CHVs are the primary healthcare system agents responsible for linking women to ANC and FP in rural communities [7], and as such are well positioned to deliver interventions that support the uptake of these services.

One such intervention may be urine pregnancy testing (UPT). Many women in Kenya rely on imprecise symptoms like missed menstrual periods to identify pregnancies. Uncertainty in pregnancy status may contribute to delays in seeking ANC and initiating a hormonal method of FP [8, 9]. Data on use of store-bought UPT in rural Kenya are limited, but anecdotal evidence from the study setting suggests UPT are too expensive for many rural women to regularly purchase. Studies in other low- and middle-income countries suggest that point of care UPT provision by CHVs is a low cost intervention that can contribute to earlier ANC initiation and FP uptake [10, 11]. However, no prior study has evaluated the impact of CHV-delivered UPT on ANC and FP uptake in Kenya. Furthermore, studies of CHV-delivered UPT in other settings provide limited data on participants’ and communities’ experiences. Thus, qualitative evidence that CHV-delivered UPT is an acceptable and feasible strategy to enhance access to ANC and FP is needed to guide the implementation of CHV-delivered UPT in Kenya, and may support similar interventions globally.

Therefore, this study aimed to determine whether CHV-delivered point of care UPT, post-test counselling, and referral to care is an acceptable and feasible strategy to increase uptake of ANC, FP, and other reproductive healthcare services, both to CHVs delivering the intervention and to women and men in the community.

## Methods

### Study setting

The pilot intervention was implemented in Port Victoria and Turbo, two rural communities in western Kenya. Port Victoria is located in Busia County, with a population of 893,681 [12]. Almost 90% of Busia’s population lives in rural areas [13], and less than 40% of the population owns a mobile phone [14]. Turbo is located in Uasin Gishu County, with a population of 1,163,186 [12]. Fifty-six percent of Turbo’s population lives in rural areas [13], and 51.4% of the population owns a mobile phone [14]. Health services in both Port Victoria and Turbo are available through the Academic Model Providing Access to Healthcare (AMPATH), an academic, public, and private partnership led by Moi University Medical School that provides comprehensive population health services to over 8 million people across 800 care sites in Western Kenya [15].

### Design of the pilot intervention

**Fig. 1:**
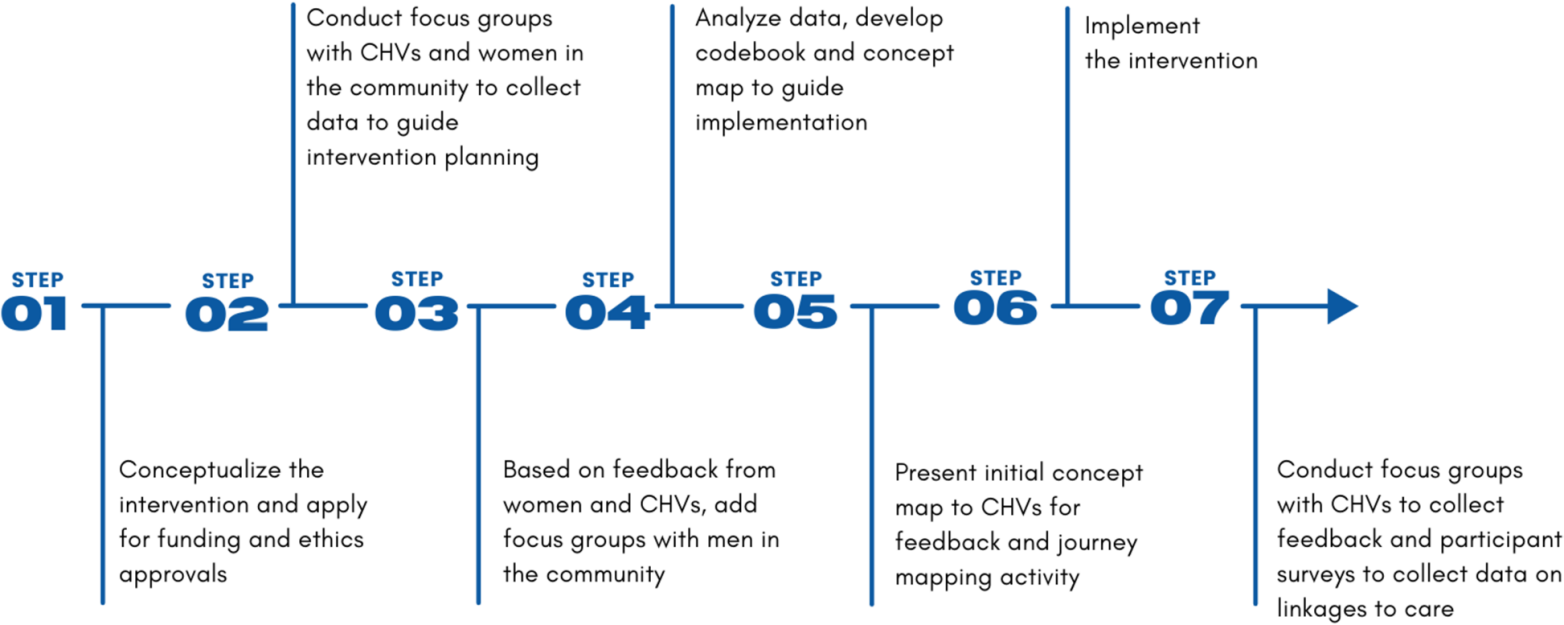
Intervention design process

We used community-based participatory methods to design, pilot, and evaluate the feasibility and acceptability of an intervention where CHVs delivered UPT, offered post-test counselling, and provided women with referrals to ANC, FP, and other reproductive healthcare services. The pilot benefited from pre-existing relationships between CHVs in the community and the research team through the established CHV maternal health program at AMPATH. The design, pilot implementation, and evaluation process is outlined below in 7 steps (Fig. 1). From July to November 2018, we conducted 12 pre-intervention focus group discussions (FGDs) with CHVs, women, and men to guide intervention planning. We analyzed data from the pre-intervention FGDs using the DEPICT model for collaborative coding developed for participatory research (Fig. 1, steps 3–6) [16]. Each transcript was read by at least two team members (CB, ACD, CK, VN, JT, and FY). The team met for two sessions to discuss the data and design a concept map and preliminary code book. The team presented the concept map to the CHVs and elicited their feedback through journey-mapping. Journey mapping is a design technique that has been used elsewhere in healthcare to explore patient experiences and engage diverse stakeholders in intervention design [17]. CHVs worked through journey maps of potential clients’ experiences accessing UPT (for example, an adolescent who is sexually active and seeking UPT without her parents knowledge, an older woman with HIV, and a young woman who had been raped) to explore issues that might guide intervention design and implementation.

### Pilot procedures

Pilot implementation took place between December 2018 and May 2019. Fifteen CHVs in each community were recruited from the established AMPATH CHV program to participate in the pilot. CHVs were informed about the aims of the research study and that as part of the study they would receive training on how to deliver UPT, time UPT appropriately after menstruation, interpret UPT results, and provide post-test counselling and referral. Training also included instruction on topics requested by CHVs, including how to provide counselling to participants who had experienced rape, stigma, sexually transmitted infections, and infertility.

CHVs informed women in the community about the opportunity to receive free point of care UPT, and offered enrolment in the study to women who wanted UPT and/or who described signs or symptoms associated with pregnancy at a CHV visit. Women who chose to enrol were contacted by the study research assistant, who explained the study procedures and obtained verbal consent over the phone. Women who declined to enrol in the study were still able to receive free UPT and post-test counselling and referral. Women who consented to enrol completed a pre-UPT survey which collected demographic information and their reasons for seeking UPT. They agreed to be contacted by study staff to complete a survey on access to care post-UPT. Quantitative data on participant demographics and outcomes are reported separately.

**Fig. 2:**
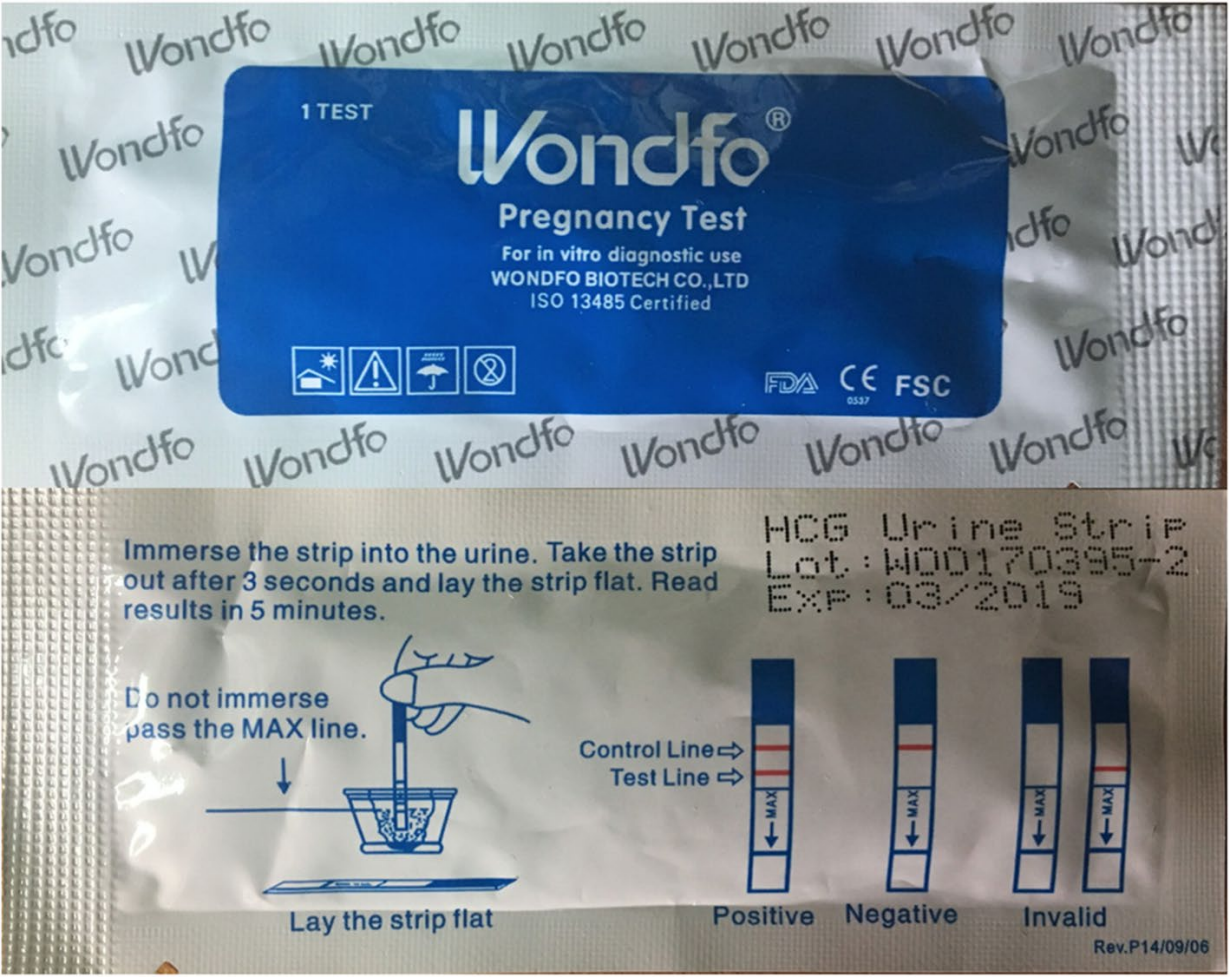
Image of the urine pregnancy test kit, front and back

CHVs provided participants with a UPT (see Fig. 2). Women were given the options of: urinating in a cup and performing the test with assistance from the CHV, or performing the test herself privately then returning to the CHV to discuss the results.

Participants were given the choice between two methods of post-test counselling: counselling from the CHV in-person, or from a study staff member by telephone. Counselling focused on linking to the appropriate reproductive health service(s), including ANC, FP, infertility care, and more. Abortion is not legal in Kenya except in certain circumstances, for example, if the life or health of the mother is in danger [18], and access to safe abortion services is limited. For information on safe, legal abortion care, CHVs were trained to refer women to a study staff via a confidential telephone number.

### Ethics & human subjects protection

This study was approved by the Moi University/Moi Teaching and Referral Hospital Institutional Review and Ethics Committee, the Indiana University Institutional Review Board, and the Kenyan National Commission for Science Technology and Innovation (NACOSTI). We also received permission to run the intervention in Busia and Uasin Gishu Counties from County Health Team leaders. Participants in the FGDs and community-based participatory activities (journey mapping, see Fig. 1) gave written informed consent. Participants who received UPT in the pilot intervention gave verbal informed consent. To protect participant privacy, no identifying information was included in transcripts during data analyses. Furthermore, study findings were shared with stakeholders, beginning with the communities who participated in the research. All methods were carried out in accordance with relevant guidelines and regulations.

### Data collection

This study includes qualitative data collected in pre-intervention FGDs (6 per location; 2 with CHVs, 2 with women, and 2 with men), and post-intervention FGDs (2 per location with CHVs who delivered the intervention). Invitations to join the FGDs were spread via announcements to CHVs and word of mouth. Each FGD included 7–9 participants recruited via convenience sampling and was moderated by experienced facilitators including the author CK. Some FGDs were observed by authors CB or JT. FGD participants were informed about the aims of the study and that their comments would contribute to implementation and evaluation of the UPT pilot. FGDs were conducted in community health centers and lasted two to three hours. Pre-intervention FGDs focused on questions about the acceptability of CHV-delivered UPT, counselling, and referral; potential barriers; acceptability and feasibility of phone-based and in-person modes of post-test counselling; and factors that might affect linkage to care post-UPT. Post-intervention FGDs explored the experiences of CHVs who delivered the intervention. FGDs were guided by a moderator using FGD guides available in Additional file 1. Discussions were held in Kiswahili and audio-recorded, then professionally transcribed and translated into English.

### Data analysis

We took a pragmatist orientation to qualitative content analysis. A pragmatist approach, as described by Patton et. al., is characterized by an emphasis on experience, outcomes of action, and shared beliefs, without defining a particular perspective on the nature of reality or truth [19]. Qualitative content analysis is a descriptive methodology for searching out, describing, and contextualizing meaning within a text [20].

Acceptability was operationalized as community members’ willingness to access UPT and CHVs’ willingness to provide UPT in a way that met the program’s aim of increasing uptake of ANC, FP, and other reproductive healthcare services. Feasibility was operationalized as community members’ ability to use UPT, and CHVs’ ability to provide UPT, in a way that met the program’s intended aim of increasing uptake of ANC, FP, and other reproductive health services. Coding focused on identifying factors that affected acceptability and feasibility. Codes representing similar ideas were collected together into themes and sub-themes and sorted by whether they affected acceptability, feasibility, or both [21].

As described above, team members (CB, ACD, CK, VN, JT, and FY) inductively coded pre-intervention FGD transcripts and developed a concept map and code book. For the post-intervention FGDs, MK coded the transcripts using the code book and developed additional inductive codes, and the team met to review codes, themes, and subthemes. MK then re-coded the pre- and post-intervention transcripts using a finalized codebook, and CK checked codes, themes, and subthemes by coding a sample of transcripts. Codes, themes, and subthemes were discussed and reviewed by the team at meetings throughout the coding process and discrepancies were resolved by discussion. MK kept reflexive memos throughout coding. Reflexive memos and discussions addressed that the authors as Canadian, Kenyan, American researchers are to varying degrees structurally and socially distant from the participants, and that biases about approaches to certain topics, for example, termination of pregnancy or reproductive healthcare for adolescents, may differ within the research team and between the research team and participants. Discussions and memos focused on attempting to stay close to the data in our descriptions and interpretation. We used NVIVO 12 software for data management. Please also see the COREQ checklist for this study included in Additional file 2.

## Results

For an overview of themes, subthemes, and key points, see Table 1.

**Table 1:** Overview of themes, subthemes, and key points

1. Factors that contributed to acceptability of the intervention
1.a. The intervention improved access to UPT and reproductive healthcare services •Home visits by CHVs eliminate costs and time spent traveling to a clinic for UPT, which is particularly beneficial for women who experience stigma in pregnancy or whose freedom to travel is limited •Early identification of pregnancy supports early initiation of ANC, and negative pregnancy tests support timely initiation of FP •CHVs support links to downstream reproductive care and act as liaisons between women and other healthcare providers
1.b. The intervention supported more informed choices about pregnancy and reproductive health, but not necessarily for women with unwanted pregnancies interested in abortion •UPT supports women’s and families’ abilities to plan for birth, for example, by saving money •UPT opens up opportunities for women to make major life choices that are affected by pregnancy status, for example, leaving a marriage •Some participants expressed concerns that women would “misuse” the information from UPT to make choices they did not think were acceptable, for example, around abortion •Some CHVs and non-CHV participants thought counselling by CHVs should be directed towards preventing abortion
1.c. Acceptability of the intervention depends on trust between CHVs and the community •CHV-delivered UPT and counselling was acceptable because CHVs were generally trusted and respected •Concerns about CHVs’ confidentiality, and that CHVs’ biases and conflicts would affect access to UPT, are barriers to acceptability •Post-UPT counselling with a study staff member is an acceptable way to address concerns about confidentiality with a CHV •CHVs themselves described a sense of great value from delivering UPT, a service which provides a diagnosis, and may have enhanced trust in CHV’s skills
**2. Factors that contributed to or detracted from the feasibility of the intervention**
2.a. It is feasible for CHVs to provide UPT, counselling, and referrals if the appropriate supplies are provided •Appropriate supplies included enough test kits, transportation or remuneration of transportation costs for CHVs, and a carrying bag
2.b. It may not always be feasible for women to access reproductive healthcare services even if a CHV successfully delivers UPT, post-test counselling, and a referral •This intervention does not address downstream barriers to accessing care at distant clinics (costs, distances, mistreatment) •Some men in the community’s negative attitudes toward FP might limit women’s access to FP services
**3. Spanning both acceptability and feasibility: adequate financial, educational, and professional support for CHVs**
3.a. The additional work involved in providing UPT, counselling, and referral exacerbated pre-existing financial strain on CHVs •CHVs live with economic insecurity, and volunteer work infringes on their time for income-generating work •UPT, which was extremely popular, exacerbated the demands on CHVs •Participants thought financial strain on CHVs could be addressed by providing financial support, as a stipend or in-kind
3.b. CHVs requested additional educational and professional supports •CHVs requested additional training for counselling around unwanted pregnancies, adolescent pregnancies, and infertility •CHVs described fears that they might be blamed for miscarriages, abortions, or breaches of confidentiality

### 1. Acceptability

#### 1.a. CHV-delivered UPT and counselling improved access to UPT and reproductive healthcare services

Participants emphasized that free provision by CHV made it easier to access UPT. Home visits by CHV, or visiting a CHV in the local area, also eliminated costs and time spent traveling to a clinic for UPT:

*To some women it is difficult to go or reach hospital so it will very helpful to have these CHVs go to them because they are close to them* (Woman, Port Victoria, Pre-intervention FGD #2)

While CHV-delivered UPT, counselling, and referral to reproductive health services was more convenient for all participants, FGD participants emphasized particular benefits for those who experience stigma in pregnancy, like younger and older women:

*Those girls who are under 18, they are ashamed of going to hospital, they can come to us in our homes to know their pregnancy status…I can send her to the health facility and I follow her up to ensure that she attends clinic.* (*CHV, Port Victoria, Pre-intervention FGD #2)*

Participants also described improved access for women with limited health education, women who want to access UPT without the knowledge of their husband or other family members, or women who for other reasons have limited freedoms. Although CHV were not trained to provide home visits only when women were home alone, some participants described that a home visit might be easier to keep private than an appointment requiring travel or funds:

*It’s good because some women are not allowed by their husbands to leave their houses, when the husbands leave for work, [then] the CHVs visit and provide counselling.* (Woman, Turbo, Pre-intervention FGD #2)

Participants agreed that early pregnancy identification supports early initiation of ANC, which otherwise might be delayed by misidentifying or missing signs or symptoms of pregnancy:

*You know some women’s menses are inconsistent. This can be confusing because she will not know whether she has conceived or not. So, it will be wise for her to go to the hospital and be tested and find out her status.* (Woman, Port Victoria, Pre-intervention FGD #1)

Participants also agreed that a negative pregnancy test supports initiation of FP, because many women avoid initiating a hormonal method of FP if they are concerned about pregnancy. At post-test counselling, CHVs can also encourage women with negative pregnancy tests who do not want to get pregnant to seek FP services as soon as possible:

*[If] she was told to go back on a certain date [for family planning] and she failed to do so, you see she will suspect herself and rush to you for help. She will say that she has missed for around 5 days and she is not sure if she is pregnant but would like to go back to family planning.* (CHV, Turbo, Post-intervention FGD #2)

CHV-delivered UPT also created opportunities to link clients to other reproductive care, including infertility care, HIV care, mother’s groups (Chamas), and to enrol in the Kenya National Health Insurance Fund. For women who might not have otherwise sought reproductive healthcare, UPT itself served as a reason to seek out the CHV, and then the CHV supported and encouraged her to access health services over follow up visits or by acting as a liaison between the woman and other healthcare providers:

*[When] there is misunderstanding between the clients and the healthcare provider…I have to liaise with my CHV because she is the one who referred me to the hospital… it is this same CHV who referred you to the hospital, and escorted you there and maybe counselled on FP side effects and [she] takes you back to the hospital maybe for the 3*^*rd*^* time to be assisted.* (Woman, Port Victoria, Post-intervention FGD #1)

#### 1.b. CHV-delivered UPT and counselling supported more informed choices about pregnancy and reproductive health, but not necessarily for women with unwanted pregnancies interested in abortion

While “knowing your status” (CHV, Port Victoria, Planning FGD #1) after UPT was a benefit by itself, UPT and counselling from a CHV also enhanced women’s and families’ abilities to plan for the future and make choices about their reproductive health. In addition to the role UPT plays in initiating ANC and FP, discussed above, positive UPT results facilitate planning for a new child, for example by saving money:

Moderator: *How will you prepare yourself [after an early positive pregnancy test]?*

Respondent: *By going to ANC, shopping for your baby, saving some money too. (Woman, Turbo, Pre-intervention FGD #2)*

Beyond choices directly about seeking reproductive healthcare or planning for a child, access to UPT opened up opportunities for women to make major life choices that depended on whether or not she was pregnant:

*For me also, when I was providing the urine pregnancy tests, there is a mother who came to me and she looked anxious…She felt so happy about the results being negative and when I prompted to know more she told me her husband was a drunkard and she never wanted to have a baby with him...I realized she came to confirm if she was pregnant or not so that she could make her decisions early enough.* (CHV, Port Victoria, Post-intervention FGD #2)

Although there were many circumstances where CHV-delivered UPT and counselling opened up opportunities for women to make choices about their reproductive health, not all choices were acceptable to all participants. Some CHVs, men, and women had concerns about women being able to access UPT without supervision from CHVs, because they could “misuse” the information to seek abortion or to hide extramarital affairs:

*Some women are deceitful, they will be testing themselves frequently because maybe they cheat.* (Male participant, Turbo, Pre-intervention FGD #2)

Many CHVs and non-CHV participants thought CHVs should try to prevent abortions, for a variety of reasons including religious beliefs, fears around unsafe abortions, and the illegality of abortion in Kenya. However, some participants did voice their support of safe abortion:

*It should be made clear to the parents and these women that the kit has not been provided for promoting ill doings like abortion but for better things like early detection of pregnancy for better and early management and treatment.* (Woman, Port Victoria, Pre-intervention, FGD #1)

*If their only option was to do abortion, then it should be a safe abortion.* (CHV, Port Victoria, Post-intervention FGD #2)

I *counselled her telling her the effects of abortion and the importance of children and she decided to keep the baby.* (CHV, Turbo, Post-intervention FGD #2)

There were also stories where a CHV conducted repeat follow up visits to provide counselling that may have been against women’s wishes, or in ways that violated women’s privacy. These stories were usually in the context of an unwanted or unplanned pregnancy. Although it was often unclear what happened during counselling from a CHV in these circumstances, sometimes (but not always or explicitly) the follow up visits may have been an effort to prevent abortion:

Respondent 1: *For the one who the pregnancy test results came positive and she has refused to be counselled, on my side if she is married you can secretly tell her husband that I would want to pay both of you a visit at a certain date… So, when you pay them a visit, you go there like a stranger and provide counselling. So, if the husband is a good man, the wife will eventually agree.*

Respondent 2: *And if you [try to counsel her] again and she refuses?*

Respondent 1: *But we have other people like the chief. We have to network. She might not listen to you but she can listen to [other CHV].* (CHV, Port Victoria, Post-intervention FGD #1)

#### 1.c. Acceptability of CHV-delivered UPT depends on trust between CHVs and the community

CHV-delivered UPT and counselling was acceptable because CHVs were generally trusted and respected. Although most participants had positive perceptions of CHVs’ ability to uphold confidentiality, some participants did express concerns about confidentiality:

Moderator: *What would be the drawbacks of getting [UPT] from a CHV?*

Respondent: *…The issue of confidentiality. The CHV tests a client then goes about disclosing the results to people.* (Man, Port Victoria, Pre-intervention FGD #1)

Biases and conflicts between CHVs and community members were perceived to potentially affect confidentiality, access to UPT, or the quality of care received from a CHV. Stigma around HIV status might also affect acceptability, given that some participants expressed concerns that UPT might be mistaken for an HIV test:

Respondent: *Some men are different [and] some will argue.*

Moderator: *Why will some argue what comes to their minds?*

Respondent: *They think the kit is used to test for a certain illness [HIV].* (Woman, Turbo, Pre-intervention FGD

We offered participants the option of choosing to have post-UPT counselling over the phone with a study staff member, which emerged as a potential alternative for people who felt uncomfortable receiving counselling in-person from a CHV. However, FGD participants also noted that phone counselling with a study staff member would be inaccessible for clients without phones or money for airtime, and could lead to a breach of confidentiality if a woman did not have private phone access. More than one CHV described clients who refused to even take the card with contact information for phone-based counselling:

Moderator: *In your opinion which mode of counselling would you prefer? Phone based or CHV?*

Respondent: *Being counselled and referred by phone, because there are some things I cannot tell the CHV.* (Woman, Turbo, Pre-intervention FGD #2)

*There are those who don’t want you to try to even call them, they don’t want their people to know.* (CHV, Turbo, Post-intervention FGD #2)

The vast majority of women and men in the FGDs expressed positive views of CHVs and their role as health leaders. CHVs themselves described a sense of great value from delivering UPT, a service which provides a diagnosis, and may have enhanced trust in CHVs’ skills. Providing UPT led to rewarding relationships with community members, and the pilot gave CHVs skills and knowledge that they could use to improve the health of their communities and their own families.

*[CHVs] are also respectful and of good morals and are up to task. They also visit and do follow-up on us and our children, whether we are sick or not. So, I can say they can be of great help to us.* (Woman, Port Victoria, Pre-intervention FGD #1)

*Whenever I was going, people referred me as a Doctor. They believed that by providing the pregnancy test services to them, then I was a doctor.* (CHV, Port Victoria, Post-intervention FGD #1)

*We would like to give [UPT] out because they were good in the community and we also got to be educated.* (CHV, Turbo, Post-intervention FGD #1)

### 2. Feasibility

We identified two distinct components of feasibility: is it feasible for CHVs to provide UPT, counselling, and referrals to care; and is it feasible to use UPT and counselling as a means to increase linkages to reproductive health care?

#### 2.a. It is feasible for CHVs to provide UPT, counselling, and referrals to care if the appropriate supplies are provided

Appropriate supplies included enough test kits for CHVs to distribute free of charge, transportation or remuneration of transport costs for CHVs (e.g. bicycles), and a carrying bag. Other supports for CHVs (including financial support) impact acceptability as well as feasibility, and are discussed in more detail below.

#### 2.b. It may not always be feasible for women to access reproductive healthcare services even if a CHV successfully delivers UPT, post-test counselling, and a referral

First, participants acknowledged that UPT, post-test counselling, and a referral would not alter downstream factors that make healthcare difficult to access, including costs at healthcare facilities, mistreatment at healthcare facilities, and distance to health facilities:

*You can refer a mother but when she goes to the [nearby clinic] she is told to go to Turbo [the further clinic]. She doesn’t have money for transport to go to Turbo.* (CHV, Post-intervention FGD #1)

Second, although men in the FGDs said that they generally approve of women accessing UPT and ANC, some men explained that they would not approve of their wives accessing FP because their religion, community, or traditions did not find FP acceptable. Many men also expressed concerns that they would experience side effects if their wives used FP. These concerns were reflected in FGDs with women, who shared concerns about FP side effects and explained that some women had to negotiate FP use with partners or seek FP without telling their partners:

*It’s good to use the UPT kit to enable me as the husband to know if my wife is pregnant for purposes of planning.* (Man, Turbo, Pre-intervention FGD #2).

*In my religion what they detest is the use of contraceptives.* (Man, Port Victoria, Pre-intervention FGD #2)

*If I can ask, since these [family planning] drugs affect women, can they affect me as well?* (Man, Port Victoria, Pre-intervention FGD #2)

*There are some husbands that are against family planning…she sometimes has to do it in secret.* (Woman, Turbo, Pre-intervention FGD #2)

### 3. Spanning both acceptability and feasibility: adequate financial, educational, and professional support for CHVs

#### 3.a. The additional work involved in providing UPT, counselling, and referral exacerbated pre-existing financial strain on CHVs

Under the current Kenya Ministry of Health Policy, CHVs receive no remuneration for their services. Participants across all FGDs described CHVs as having a large volume of work without sufficient financial support:

*For instance, in our place in XXX Unit we have 1 CHV…villages are densely populated, so you find that there is always a high demand…this is overwhelming if it is just one [CHV] rendering the services.* (Woman, Port Victoria, Pre-intervention FGD #1)

They emphasized that CHVs often live with economic insecurity, and volunteer work infringes on CHVs’ time for income-generating work:

*You will find that some of these CHVs have their other personal engagements [work] so that sometimes when they are needed it can become difficult due to commitments to earn daily bread.* (Man, Port Victoria, Pre-intervention FGD #1)

UPT was extremely popular and exacerbated the demands on CHVs. In addition to time spent delivering UPT, CHVs did more follow-up visits post-UPT to ensure clients accessed referral services, which compounded their pre-existing challenges with out-of-pocket expenses, including travel to home visits and phone airtime to talk to clients:

*They call and come to your door, you will even find them at the door waiting for you if you had gone somewhere.* (CHV, Turbo, Post-intervention FGD #1)

*You keep revisiting that person wanting to know how she is, if she has gone to the clinic. In that revisiting you have no airtime, you have no money to get on a motorcycle.* (CHV, Turbo, Post-intervention FGD #2)

CHVs and non-CHV participants thought that financial strain on CHVs could be addressed by providing CHV with financial support, as a stipend or in-kind (e.g. food, a bicycle):

*If possible allocate to them some stipend to motivate them…at the end of the day they will get some soap at least.* (Man, Port Victoria, Pre-intervention FGD #2)

#### 3.b. CHVs requested additional educational and professional supports

Delivering UPT and counselling placed CHVs in challenging situations, and many CHVs asked for additional training on how to counsel around unwanted pregnancies, adolescent pregnancies, and infertility.

*Referring this woman was very hard because she didn’t want to, she had not expected to be pregnant. So, it was hard for me, it took a long time because she needed advanced counselling yet you are not a counsellor.* (CHV, Turbo, Post-intervention FGD #1)

CHVs also described fears that they might be blamed for miscarriages, abortions, and breaches of confidentiality, or accused of stealing UPT:

*If her husband sees her with that kit and knows she is using it then maybe she tested positive for pregnancy then he later discovers that she is no longer pregnant, what will be his perception about me [the CHV], and what would follow after that?* (CHV, Turbo, Pre-intervention FGD #1)

Finally, CHVs discussed the challenges of being the person with the most healthcare qualifications in the local area and often the default first responder, despite the limits of their training:

*Some of us we do not know what to do. When a mother is gasping for air when breathing…and the next day the [clinic] is not working.* (CHV, Port Victoria, Post-intervention FGD #2)

## Discussion

We found that CHV-delivered point of care UPT, post-test counselling, and referral to care is an acceptable and feasible strategy to increase uptake of ANC, FP, and other reproductive healthcare services, from the perspectives of CHVs delivering the intervention and women and men in the community. Factors that contributed to the intervention’s acceptability were: (1) CHV-delivery made UPT more accessible; (2) UPT and counselling supported women and men to build knowledge and make informed choices, although not necessarily for women with unwanted pregnancies interested in abortion; (3) CHVs were generally trusted to provide counselling, and alternative counselling providers were available according to participant preference. A factor that enhanced the feasibility of CHV delivering UPT and counselling was access to appropriate supplies (e.g. a carrying bag). However, factors that detracted from the feasibility of women actually accessing referral services after UPT and counselling included (1) downstream barriers to accessing referral services, and (2) some male community members’ negative attitudes toward FP. Finally, improved financial, educational, and professional supports for CHVs would be needed to make the intervention acceptable and feasible in the long-term.

CHVs and community members described CHVs providing UPT, counselling, and referral to care as highly acceptable in part because CHVs are accessible at a home visit or in the village. Accessibility is consistently identified as a benefit of many types of CHV-delivered interventions [22–24], including a previous trial of CHV-delivered UPT in Nepal [11]. The added value of our qualitative analysis is that we clearly elucidate why accessible UPT matters—beyond being more convenient, when CHVs provide UPT followed by counselling, they encourage and support women who might delay accessing health services because of time and resource constraints or stigma. We also found evidence that ongoing CHV involvement, including accompaniment to clinic visits, supports women who face barriers once they reach the health facility.

Our findings are in some respects aligned with previous literature that positions UPT as a tool to support informed reproductive health choices, like initiating ANC, starting hormonal FP, or planning for a new child [10, 11, 25]. However, we also found that some CHVs offered value laden counselling around unwanted pregnancies and abortion, which has not been reported in previous CHV-delivered UPT interventions. Abortion in Kenya is not legal except under certain circumstances, and is highly stigmatized, and safe abortion services can be difficult to access [26–29]. In our study, CHVs could refer women to study staff to discuss options for safe abortion. Although some CHVs did refer women to study staff to discuss safe abortion, our data also suggest that some CHVs tried to convince women not to seek abortions in ways that may have breached women’s confidentiality and impeded access to safe abortion services. Additionally, CHVs' actions to try to prevent women from having abortions were acceptable, or even preferable, within the context of some participants’ views. This finding supports evidence that CHVs’ ability (and we contend, willingness) to shift health behaviors depends on sociocultural norms [30], and raises questions about what happens on the ground when what is acceptable to program designers differs from what is acceptable to CHVs and the broader community. CHV-delivered UPT programs in Kenya will have to address that ‘acceptable’ management of unwanted pregnancies will mean different things to women, CHVs, funders, healthcare providers, and policy makers, and reconcile potentially conflicting notions about what constitutes appropriate counselling.

Our findings echo other studies which have found that strong, trusting relationships between CHVs and community are essential to the acceptability of CHV-delivered interventions [31–35]. As in other studies, some community members expressed concerns about CHVs’ confidentiality [24], potentially ameliorated by providing the option of phone counselling with a study staff member. Enabling CHVs to provide UPT and post-test counselling builds trust and respect for CHVs in the community, which may bolster CHVs’ role, as outlined in the Kenya Community Health Policy, in strengthening links between communities and the health system [7].

Although participants agreed that it was feasible for CHVs to distribute UPT and provide counselling and referrals to care with appropriate supports (e.g. carrying bags), it was not as clear that it would be feasible for women in the community to actually access referral services. Access to UPT does not address downstream barriers to healthcare access like distances to clinics, costs, and quality of care at clinics. Additionally, men’s views are important contextual factors in determining whether CHV-delivered interventions for women can actually increase health service use [30]. Our experience conducting focus groups with male community members prior to the intervention allowed us to address some of their concerns during implementation, and reinforces evidence that involving men can improve uptake of reproductive health services [34, 36, 37].

Finally, appropriate financial, educational, and professional supports for CHVs emerged as a major question in the longer-term sustainable acceptability and feasibility of this intervention, and CHV-delivered interventions more broadly. As in numerous other studies, we found that volunteer work imposed profound financial and time burdens on CHVs [23, 31, 38, 39], which were exacerbated by also delivering free UPT. A growing body of literature on the experiences of CHVs has raised serious concerns about volunteerism, suggesting that volunteer duties may worsen CHVs’ pre-existing poverty [38], and contribute to overwhelming work and financial insecurity [23, 31, 40–43]. These studies, and our data, raise the question of whether CHV programs exploit the volunteer labor of people living in poverty, disproportionately women, to make the health system function [38, 39]. However, the unfair burdens of volunteerism exist alongside evidence, from our study and others, that CHVs perceive great value from their community service [31, 35, 41–43], and express willingness to volunteer under financial strain while perceived or potential benefits outweigh the harms [38, 41].

Ultimately, the tension between the burdens on CHVs, and the fact that CHVs overwhelmingly wanted the intervention to continue, suggests the limits of evaluating the acceptability and feasibility of a CHV-delivered intervention by talking to only CHVs and community members who must make trade-offs within a system where they have limited decision-making power. How CHV programs are designed and rolled out and whose interests are considered is an urgent area for future study, using participatory methods where CHVs engage directly with government actors, healthcare-decision makers, and national and international funders who set the parameters under which CHVs volunteer.

This study has a number of limitations. First, we did not include male and female community members in our post-intervention FGDs due to time and budget constraints, and are limited in our ability to draw conclusions about how community members experienced the intervention. Second, our insights are based within a particular context, and some findings may not apply in other settings or other CHV programs. Third, no CHVs or community members were directly involved in data coding or manuscript writing, which means that our interpretations are subject to our biases and positionality as Canadian, Kenyan, and American researchers, clinicians, and students, who are (to varying degrees) socio-economically, culturally, and in other many other ways distant from participants. Finally, FGD participants may have been subject to social desirability bias, or biased by a desire not to have UPT taken away.

Our study also has a number of strengths. We used established community-based participatory research strategies to guide the development of the intervention and data analysis [16]. We were able to include a relatively large number of participants, including male community members, in our focus groups. Our study is also grounded in an established CHV program and long-term relationships with CHVs and communities, which may have helped participants feel comfortable speaking honestly. In addition, the research team is composed of both clinicians and researchers from Kenya, Canada, and the United States through the well-established AMPATH collaboration; the team collaborated extensively throughout the entirety of the project. Finally, we are the first to perform in-depth qualitative interviews with three groups of stakeholders in a CHV-delivered UPT intervention, which provides rich information to guide future program development.

## Conclusion

Free or low-cost UPT, counselling, and referral to reproductive health services delivered by CHVs in rural communities in Kenya was acceptable and feasible in this context, and may support early initiation of ANC and uptake of FP. Additional qualitative work is needed to explore implementation challenges, including issues related to unwanted pregnancies and abortion, the financial burden of volunteerism on CHVs, and educational and professional supports for CHVs.

## Supplementary Information

**Additional file 1. **Focus group discussion guides.

**Additional file 2.** COREQ (COnsolidated criteria for REporting Qualitative research) Checklist.

## Data Availability

The datasets generated and/or analysed during the current study are not publicly available because they contain information that could compromise participant privacy and confidentiality but are available from the corresponding author on reasonable request.

## Abbreviations

AMPATH: Academic Model Providing Access to Healthcare
ANC: Antenatal care
CHU: Community health units
CHV: Community health volunteer
FGD: Focus group discussion
FP: Family planning
HIV: Human Immunodeficiency Virus
NACOSTI: Kenyan National Commission for Science Technology and Innovation
UPT: Urine pregnancy testing
WHO: World Health Organization
