# Supplementary material for: Acceptability and feasibility of community-based provision of urine pregnancy tests to support linkages to reproductive health services in Western Kenya: a qualitative analysis
Source: BMC Pregnancy Childbirth. 2022 Sep 1;22:674. doi: 10.1186/s12884-022-04869-8 (PMC9434878; doi:10.1186/s12884-022-04869-8)
Supplement: Supplementary file 1 — Additional file 1. Focus group discussion guides. [file 12884_2022_4869_MOESM1_ESM.pdf]

Additional File 1

Pre-intervention focus group discussion guide for Community Health Volunteers (Kiswahili)

Pre Intervention Focus Group Guide – Madaktari wa vijiji

- **Swali la kuchunguza:** Je! Ni jinsi gani Madaktari wa vijiji wanaweza kuwasaidia wanawake kutambua mimba na kuwasaidia kupata huduma za afya?

Vidokezo:____

- **Maswali ya kufuatilia:** Ungejisikia vizuri kupeana vifaa vya kupima ujauzito?

Vidokezo:____

- Je! Kutakua na faida gani kutokana na kupeana vifaa vya kupima ujauzito ?

Vidokezo:____

- Je! Ni ugumu gani unaoweza kukumbuna nao katika kupeana vifaa vya kupima ujauzito?

Vidokezo:____

- Je! Ni mafunzo gani ya ziada ambayo yanaweza kukusaidia kupeana vifaa?

Vidokezo:____

Additional File 1

Pre-intervention focus group discussion guide for Community Health Volunteers (Kiswahili)

- **Swali la uchunguzi:** Je! Ungejisikia vizuri kuwashauri wanawake kuhusu nini cha kufanya ikiwa watapimwa na matekeo kuonyesha kuwa wana ujauzito?

Vidokezo:____

- **Swali la kufuatilia:** Je! Ungewashauri vipi wanawake ikiwa matekeo yao yangeonyesha kuwa wana ujauzito?

Vidokezo:____

Je, ungewauliza vipi jinsi walivyohisi kuhusu kuwa mjamzito?

Vidokezo:____

Je! Ungewashauri vipi ikiwa hawakufurahishwa na ujauzito huo?

Vidokezo:____

Je! Ungewaelekeza wapi iwapo matokeo ya yangeonyesha kua wana ujauzito?

Vidokezo:____

Je! Ni mafunzo gani ya ziada yanaweza kukusaidia kutoa ushauri kuhusu matekeo ambayo yanaonyesha kuwa kuna ujauzito?

Additional File 1

Pre-intervention focus group discussion guide for Community Health Volunteers (Kiswahili)

- **Vidokezo:**____
- **Swali la kuchunguza:** Je! Ungejisikia vizuri kuwashauri wanawake kuhusu nini cha kufanya ikiwa matokeo yangeonyesha kuwa hawana ujauzito?

Vidokezo:____

- **Swali la kufuatilia:** Je! Ungewashauri vipi wanawake ikiwa matekeo yao yangeonyesha kuwa hawana ujauzito?

Vidokezo:____

Je! Ungewauliza vipi jinsi walivyohisi kuhusu kutokuwa na ujauzito?

Vidokezo:____

Je! Ungewashauri vipi ikiwa hawataki kuwa na mimba?

Vidokezo:____

Je! Ungejisikia vizuri kuwashauri wanawake kuhusu kutumia mbinu ya kupanga uzazi?

Vidokezo:____

____
____
____

Je! Ungewaelekeza wapi kama wangetaka kutumia mbinu ya kupanga uzazi?

Vidokezo:____
____
____
____
____

Je! Ungewashauri vipi kama wangetaka kuwa na ujauzito?

Vidokezo:____
____
____
____
____

Ungewaelekeza wapi kama wangetaka kuwa na ujauzito?

Vidokezo:____
____
____
____
____

Je! Ni mafunzo gani ya ziada ambayo yanaweza kukusaidia kutoa ushauri nasaha kwa matokeo ambayo yanaonyesha kuwa hana ujauzito?

Vidokezo:____
____
____
____
____

Je! Unafikiria nini kuhusu kuwapa wanawake nambari ya simu ili wapate ushauri wa kibinafsi kupitia ujumbe au kupigiwa simu?

- **Vidokezo:**____
  ____
  ____
  ____
  ____
  ____
- **Swali ya kumalizia:** Je! Kuna kitu kingine chochote unachotaka tujue kuhusu kupeana vifaa vya kupima ujauzito ?

Additional File 1

Pre-intervention focus group discussion guide for Community Health Volunteers (Kiswahili)

Vidokezo:____
____
____
____
____

Je! Kuna kitu kingine chochote unachotaka tujue kuhusu kutoa ushauri nasaha na rufaa kwa wanawake ambao ni wajawazito?

Vidokezo:____
____
____
____
____

Je! Kuna kitu kingine chochote unachotaka tujue kuhusu kutoa ushauri nasaha na rufaa kwa wanawake amabao si wajawazito?

Vidokezo:____
____
____
____
____

Additional File 1

Pre-intervention focus group discussion guide for women (Kiswahili)

Pre Intervention Focus Group Guide – Wanawake

- **Swali la kuchunguzi:** Unajuaje unapokuwa mjamzito?
- **Vidokezo:**____

____

____

____

____

- **Swali la kufuatilia:** Je! Ni wakati gani unaweza kutumia vifaa vya kupima ujauzito?
- **Vidokezo:**____

____

____

____

____

Je! Ungeipata wapi?

- **Vidokezo:**____

____

____

____

____

Je! Una matatizo gani katika kupata au kutumia vifaa ivyo?

Vidokezo:____

____

____

____

____

Una maoni gani kuhusu kuipata kutoka kwa madaktari wa vijiji?

Vidokezo:____

____

____

____

____

Je! Kuna faida gani kuipata kutoka kwa madaktari wa vijiji?

Vidokezo:____

____

____

____

____

Je, ni vikwazo gani vya kupata kutoka kwa madaktari wa vijiji?

Additional File 1

Pre-intervention focus group discussion guide for women (Kiswahili)

- **Vidokezo:**____
- **Swali la kuchunguza:** Unajauje cha kufanya iwapi matokeo yataonyesha kuwa una ujauzito?

Vidokezo:____

- **Swali la kufuatilia:** Ungeenda wapi ikiwa matokeo yangeonyesha kuwa una ujauzito?
- **Vidokezo:**____

Ungefanya nini ikiwa huna furaha kuhusu kuwa mjamzito?

Vidokezo:____

Je, ni wakati gani wa ujauzito unaweza kwenda kwenye kituo cha afya?

Vidokezo:____

Je, ni faida gani za kwenda kliniki ya ujauzito mapema katika ujauzito wako?

Vidokezo:____

Una maoni gani kuhusu kupata ushauri nasaha na rufaa kutoka kwa daktari wa kijiji?

Additional File 1

Pre-intervention focus group discussion guide for women (Kiswahili)

Vidokezo:____

Una maoni gani kuhusu kupata ushauri wa kipekee na rufaa kupitia kwa ujumbe au kupigiwa simu?

- **Vidokezo:**____
- **Swali la kuchunguza:** Ungefanya nini ikiwa kipimo kilionyesha hauna ujauzito na pia haukua unataka kua mjamzito?
- **Vidokezo:**____
- **Swali la kufuatilia:** Je! Ungetafuta mbinu ya kupanga uzazi?
  Vidokezo:____

Je! Ungetafuta mbinu ya kupanga uzazi wapi?

Vidokezo:____

Je! Ungekumbana na matatizo gani katika kupata mbinu za kupanga uzazi?

Vidokezo:____

Additional File 1

Pre-intervention focus group discussion guide for women (Kiswahili)

Una maoni gani kuhusu kupata ushauri nasaha na rufaa ya kliniki ya kupanga uzazi kutoka kwa daktari wa kijiji?

Vidokezo:____

Una maoni gani kuhusu kupata ushauri nasaha na rufaa kupitia ujumbe ama kupigiwa simu?

- **Vidokezo:**____

- **Maswali ya kumalizia:** Je! Kuna kitu kingine chochote unachotaka unachotaka tujue kuhusu kupokea vifaa vya kupima ujauzito kutoka kwa daktari wa kijiji?
  - **Vidokezo:**____

Je! Kuna kitu chochote unachotaka tujue kuhusu kupokea ushauri nasaha na rufaa kutoka kwa madaktari wa vijiji?

Vidokezo:____

Je! Kuna kitu kingine chochote unachotaka tujue kuhusu kupokea ushauri nasaha na rufaa kupitia kwa njia ya ujumbe au simu ya faragha?

Vidokezo:____

Additional File 1

Pre-intervention focus group discussion guide for men (Kiswahili)

Pre Intervention Focus Group Guide – Wanaume

- **Swali la kuchunguza:** Unajuaje mkeo akiwa mjamzito?

- **Vidokezo:**____
____
____
____
____

- **Maswali ya kufuatilia:** Je! Unajua kifaa cha kupima ujauzito ni nini?

- **Vidokezo:**____
____
____
____
____

- Je! Mke wako uwa anatumia kifaa cha kupima ujauzito ?

Vidokezo:____
____
____
____
____

- (Iwapo ndio) Je! Uwa anatumia wakati gani kifaa cha kupima ujauzito?

Na ni sababu gani ungemfanya kutumia kifaa iki?

Vidokezo:____
____
____
____
____

(Iwapo La) Ni kwa nini hasitumie kifaa iki?

Vidokezo:____
____
____
____
____

Je! Una maoni gani kuhusu matumizi ya kifaa cha kupima ujauzito?

Additional File 1

Pre-intervention focus group discussion guide for men (Kiswahili)

Vidokezo:

Je! Ni nini mtazamo wa wanaume katika jamii hii kuhusu madaktari wa vijiji?

Vidokezo:

Je! wanaume kutoka hii jamii wana maoni gani kuhusu madaktari wa vijiji kuwapa huduma kwa wanawake wao?

Vidokezo:

Je! Wanaume katika jamii hii wana maoni gani kuhusu madktari wa vijiji au wahudumu wa afya kuhusu suala la usiri wakati wa kutoa huduma kwa wake zao na wanajamii?

Vidokezo:

Je! Unapata faida gani kutoka kwa madaktari wa vijiji?

Vidokezo:

Je! Una matatizo gani na madaktari wa vijiji?

Vidokezo:

Je! Ni faida gani za kupata kifaa ya kupima ujauzito kutumia mkojo kutoka kwa daktari wa kijiji?

Additional File 1

Pre-intervention focus group discussion guide for men (Kiswahili)

Vidokezo:____
____
____
____
____

Je! Ni vikwazo gani ambayo inatokana na kupata kutoka kwa daktari wa kijiji?

Vidokezo:____
____
____
____
____

Je! Unaweza kufikiria kumnunulia mkeo kifaa ya kupimia ujauzito ?

Vidokezo:____
____
____
____
____

Ni wapi utanunua au kupata kifaa ya kupimia ujauzito ?

Vidokezo:____
____
____
____
____

Je! Imani yako ya kidini inaweza kuathiri hisia zako kuhusu upimaji wa ujauzito?

(Iwapo ndio): kivipi, au kwa njia zipi? Na katika njia ya kupanga uzazi?

Vidokezo:____
____
____
____
____

Je! Imani yako na mtazamo ya kitamaduni inaweza kuathiri hisia zako kuhusu upimaji wa ujauzito?

(Iwapo ndio): kivipi, au kwa njia zipi? Na katika njia ya kupanga uzazi?

Additional File 1

Pre-intervention focus group discussion guide for men (Kiswahili)

Vidokezo:____

____

____

____

____

- **Swali la kuchunguza:** Je! Ungefanya nini iwapo matokeo ingeonyesha kuwa ana ujauzito? au ungejuaje kitu la kufanya iwapo matokeo ingeoyesha kuwa ana ujauzito?
- **Vidokezo:**____
____
____
____
____
____
- **Maswali ya kufuatilia:** Je! Ungempeleka wapi iwapo matokeo ingeonyesha kuwa ana ujauzito?
- **Vidokezo:**____
____
____
____
____
____

Je! Ungempendekeza aende wapi ikiwa kipimo chake cha ujauzito inaonyesha kwamba ana ujauzito?

Vidokezo:____

____

____

____

____

Ungefanya nini kama wanandoa ikiwa hamna furaha kuhusu ujauzito?

Vidokezo:____

____

____

____

____

Mungeenda wapi? Au mungeenda kwa nani?

(Iwapo ndio): Toa sababu

(Iwapi La): Toa sababu.

Additional File 1

Pre-intervention focus group discussion guide for men (Kiswahili)

Vidokezo:____
____
____
____
____

Ungeenda wapi kwa huduma za utunzanji wa ujauzito (ANC)?

Vidokezo:____
____
____
____
____

Je! Ni ugumu gani ambazo zipo katika kupata huduma za utunzanji wa ujauzito?

Vidokezo:____
____
____
____
____

Faida gani zipo katika kupata huduma za utunzanji wa ujauzito?

Vidokezo:____
____
____
____
____

Je! Ni baadhi ya matatizo gani yanayowakabili wanawake wakati wa ujauzito?

Vidokezo:____
____
____
____
____

Je! Ikiwa matokeo inaonyesha kuwa hauna ujauzito…na hutaki mtoto mwingine? Ungeenda wapi au ungefanya nini? Au hutaki mtoto mwingine?

- **Vidokezo:**____
  ____

Additional File 1

Pre-intervention focus group discussion guide for men (Kiswahili)

____
____
____
____

- **Swali la kuchunguza:** Je! Una maoni gani kuhusu njia za kupanga uzazi na matumizi yake?
- **Vidokezo:**____
____
____
____
____
- **Maswali ya kufuatilia:** Je! Ni faida gani za kutumia njia za kupanga uzazi?
- **Vidokezo:**____
____
____
____
____

Je! Ni vikwazo ua hatari gani za kutumia njia za kupanga uzazi?

Vidokezo:____
____
____
____
____

Je! Una hisia gani kuhusu mke wako kutumia njia ya kupanga uzazi?

Vidokezo:____
____
____
____
____

Je! Wanaume katika jamii yako wana maoni gani kuhusu mbinu mahususi za kupanga uzazi? (vipandikizi, IUCDs, Depo, kondomu, BTL)

Vidokezo:____
____
____
____
____

- **Swali la kuchunguza:** Je! Unaweza kuambatana na mke wako kwenda kliniki?
- **Vidokezo:**____
____
____
____
____
____

Additional File 1

Pre-intervention focus group discussion guide for men (Kiswahili)

- **Maswali ya kufuatilia:** Faida zake ni zipi?
- **Vidokezo:**____

  Vikwazo vingekuwa vipi?
- **Vidokezo:**____
- **Swali la kuchunguza:** Je! Wana maoni gani kuhusu ushauri wa madaktari wa vijiji?
- **Vidokezo:**____
- **Maswali ya kufuatilia:** Je! Wana maoni gani kuhusu ushauri nasaha unaotegemea simu?
- **Vidokezo:**____

  Je! Kuna kitu kingine chochote unachotaka tufahamu kuhusu kupima ujauzito kwenye mkojo, matumizi yake na madaktari wa vijiji kuwapa wake zenu?
- **Vidokezo:**____
- **Maswali ya kumalizia:** Eleza uingiliaji kati tunaopanga (Kupima ujauzito)-Uliza ni nini maoni yao kuhusiana na mradi huu?

Vidokezo:____

Faida ya huu mradi ni zipi?

Vidokezo:____

Additional File 1

Pre-intervention focus group discussion guide for men (Kiswahili)

____
____
____

Hatari au vikwazo ni gani?

Vidokezo:____
____
____
____
____

Je! Tunapaswa kuondoa nini kutoka kwa huu mradi?

Vidokezo:____
____
____
____
____

Je! Ni mapendekezo gani yako kwa huu mradi katika siku zijazo?

Vidokezo:____
____
____
____
____

Additional File 1

Post-intervention focus group discussion guide for Community Health Volunteers (Kiswahili)

Post Intervention Focus Group Guide – Madaktari wa Vijiji

- **Swali la kuchunguza:** Je! uzoefu wako ulikuwa vipi katika kutoa vifaa vya kupima ujauzito ?
- **Vidokezo:**__________
- **Maswali ya kutuatilia**: Ulijisikia raha kiasi gani kutoa vifaa vya kupima ujauzito?__________

Je! baadhi ya mifano ya wakati ulijisikia vibaya ni gani (au haukujisikia vizuri) kutoa vifaa vya kupima ujauzito?

Vidokezo:__________

Je! Unafikiri ni kwa nini hakukuwa na washiriki walioripoti kubakwa?

Vidokezo:__________

Je, ulipata faida gani kwa kupeana vifaa vya kupima ujauzito ?

Vidokezo:__________

Wanawake walipata faida gani?

Vidokezo:__________

Je! ni matatizo gani ulikumbana nayo katika kutoa vifaa vya kupima ujauzito ?

Additional File 1

Post-intervention focus group discussion guide for Community Health Volunteers (Kiswahili)

Vidokezo:____

Wanawake walikabili matatizo gani?

Vidokezo:____

Je, ungependa kuendelea kutoa vifaa vya kupima ujauzito kwenye mkojo? Kwa nini ndio au kwa nini la?

- **Vidokezo:**____
- **Probe Question**: Uzoefu wako ulikuwa vipi katika kushauri na rufaa kwa wanawake kuhusu nini cha kufanya ikiwa vipimo vyao yangeonyesha kuwa wana ujauzito?
- **Vidokezo:**____
- **Follow-up Questions:** Ulijisikia raha kiasi gani kuhusu ushauri na rufaa kwa wanawake ambao vipimo vyao ingeonyesha kuwa wana ujauzito?

Vidokezo:____

Ulijisikia raha kiasi gani kushauri na kuwapa rufaa wanawake ambao hawakufurahishwa na kuwa wajawazito?

Vidokezo:____

Additional File 1

Post-intervention focus group discussion guide for Community Health Volunteers (Kiswahili)

Je, uzoefu wako wa ushauri na ushauri na rufaa kwa wanawake ambao walisema walitaka uavyaji mimba ulikuwaje?

Vidokezo:____

Je! ni faida gani ulizopata katika kutoa ushauri na kuwaelekeza wanawake ambao vipimo vyao ilionyesha kuwa wana ujauzito?

Vidokezo:____

Wanawake walipata faida gani?

Vidokezo:____

Je! ni matatizo gani ulikumbana nayo katika kutoa ushauri nasaha na rufaa kwa wanawake ambao vipimo vyao ilionyesha kuwa wana ujauzito?

Vidokezo:____

Wanawake hao walikabili matatizo gani?

Vidokezo:____

Je! ungependa kuendelea kutoa ushauri na rufaa kwa wanawake ambao vipimo vyao yanaonyesha kuwa wana ujauzito?

- **Vidokezo:**____

Post-intervention focus group discussion guide for Community Health Volunteers (Kiswahili)

____
____

- **Swali la kuchunguza**: Uzoefu wako ulikuwa vipi katika kushauri na kuwapa rufaa wanawake kuhusu nini cha kufanya ikiwa vipimo vyao inaonyesha kuwa hawana ujauzito?
- **Vidokezo:**____
____
____
____
____
____
- ***Maswali ya kufuatilia:*** Ulijisikia raha kiasi gani kuhusu ushauri na kuwapa rufaa wanawake kuhusu nini cha kufanya ikiwa matokeo yao yangeonyesha kuwa hawana ujauzito?

Vidokezo:____
____
____
____
____

Je, ulijisikia raha kiasi gani kupata ushauri na kuwaelekeza wanawake ambao hawakutaka kuwa wajawazito?

Vidokezo:____
____
____
____
____

Je, ulijisikia raha kiasi gani kupeana ushauri na kuwaelekeza wanawake waliotaka kuwa wajawazito?

Vidokezo:____
____
____
____
____

Je, ulipata faida gani katika kutoa ushauri na kuwaelekeza wanawake ambao vipimo vyao vilionyesha kuwa hawana ujauzito?

Vidokezo:____
____
____
____
____

Wanawake walipata faida gani?

Additional File 1

Post-intervention focus group discussion guide for Community Health Volunteers (Kiswahili)

Vidokezo:____
____
____
____
____

Je! ni matatizo gani ulikumbana nayo katika kutoa ushauri na rufaa kwa wanawake ambao vipimo vyao vilionyesha kuwa hawana ujauzito?

Vidokezo:____
____
____
____
____

Wanawake hao walikabili matatizo gani?

Vidokezo:____
____
____
____
____

Je! ungependa kuendelea kutoa ushauri na rufaa kwa wanawake ambao vipimo vyao vimeonyesha kuwa hawana ujauzito?

- **Vidokezo:**____
  ____
  ____
  ____
  ____
  ____
- **Swali la kuchunguza:** Je! Kuna wakati ambapo hukutoa ushauri ya baada ya matokeo ya kupimwa na rufaa?

Vidokezo:____
____
____
____
____

- **Maswali ya kufuatilia:** Ni ipi baadhi ya mifano ya lini na kwa nini hukutoa ushauri wa baada ya matokeo ya kupimwa?

Vidokezo:____
____
____
____
____

Additional File 1

Post-intervention focus group discussion guide for Community Health Volunteers (Kiswahili)

Je, uliwapaje wanawake chaguo la ushauri nasaha kwa njia ya simu?

Vidokezo:____

____

____

____

____

Unadhani ni kwa nini washiriki walichagua ushauri kwa njia ya simu?

Vidokezo:____

____

____

____

____

Je! Unafikiri ni kwa nini wanawake waliochagua ushauri kwa njia ya simu hawakuwahi kuwasiliana na msaidizi wa utafiti? Je, uliwapatia kadi ili wawasiliane na msaidizi wa utafiti?

Vidokezo:____

____

____

____

____

Je, ulihisi kuwa walihitaji ushauri nasaha unaotegemea daktari wa kijiji pamoja na ushauri wao wa kutegemea simu?

- **Vidokezo:**____

  ____

  ____

  ____

  ____
- ***Maswali ya kumalizia***: Je! kuna kitu kingine chochote unachotaka tujue kuhusu jinsi ya kubadilisha kuboresha njia ya kutoa vifaa vya kupima ujauzito katika siku zijazo?

Vidokezo:____

____

____

____

____

Je, kuna kitu kingine chochote unachotaka tujue kuhusu jinsi ya kubadilisha au kuboresha mchakato wa kutoa ushauri na rufaa kwa wanawake ambao ni wajawazito katika siku zijazo?

Vidokezo:____

____

____

Additional File 1

Post-intervention focus group discussion guide for Community Health Volunteers (Kiswahili)

____
____

Je! kuna kitu kingine chochote unachotaka tujue kuhusu jinsi ya kubadilisha au kuboresha mchakato wa kutoa ushauri nasaha na rufaa kwa wanawake ambao si wajawazito katika siku zijazo?

Vidokezo:____
____
____
____
____

Additional File 1

Pre-intervention focus group discussion guide for Community Health Volunteers (English)

Pre Intervention Focus Group Guide – Community Health Volunteers

- **Probe Question:** How can CHVs help women identify pregnancies and help them get care?

Notes: ____
____
____
____
____

- **Follow-up Questions:** Would you feel comfortable providing urine pregnancy kits?

Notes: ____
____
____
____
____

What benefits would there be from providing urine pregnant kits?

Notes: ____
____
____
____
____

What difficulties would you face in providing urine pregnancy kits?

Notes: ____
____
____
____
____

What additional training would be helpful for you to provide kits?

Notes: ____
____
____
____
____

- **Probe Question:** Would you feel comfortable counselling women about what to do if they have a positive pregnant test?

Notes: ____
____
____

Additional File 1

Pre-intervention focus group discussion guide for Community Health Volunteers (English)

- **Follow-up Questions:** How would you counsel women if they had a positive pregnancy test?

Notes:

How would you ask them how they felt about being pregnant?

Notes:

How would you counsel them if they were unhappy about being pregnant?

Notes:

Where would you refer them if they have a positive pregnant test?

Notes:

What additional training would be helpful for you to provide counselling for a positive pregnancy test?

Notes:

- **Probe Question:** Would you feel comfortable counselling women about what to do if they have a negative pregnancy test?

Additional File 1

Pre-intervention focus group discussion guide for Community Health Volunteers (English)

Notes: ____
____
____
____
____

- **Follow-up Questions:** How would you counsel women if they have a negative pregnancy test?

Notes: ____
____
____
____
____

How would you ask them how they felt about not being pregnant?

Notes: ____
____
____
____
____

How would you counsel them if they did not want to be pregnant?

Notes: ____
____
____
____
____

Would you feel comfortable counselling women about using FP?

Notes: ____
____
____
____
____

Where would you refer them if they wanted to use FP?

Notes: ____
____
____
____
____

How would you counsel them if they wanted to be pregnant?

Notes: ____________________

Where would you refer them if they wanted to be pregnant?

Notes: ____________________

What additional training would be helpful for you to provide counselling for a negative pregnancy test?

Notes: ____________________

What would you think about providing women with a phone number for them to receive private counselling by SMS or phone call?

Notes: ____________________

- **Exit Questions:** Is there anything else you want us to know about providing urine pregnancy test kits?

Notes: ____________________

Is there anything else you want us to know about providing counselling and referral for women who are pregnant?

Notes: ____________________

Is there anything else you want us to know about providing counselling and referral for women who are not pregnant?

Notes: ____
____
____
____
____

Additional File 1

Pre-intervention focus group discussion guide for women (English)

Pre Intervention Focus Group Guide – Women

- **Probe Question:** How do you know when you're pregnant?

Notes: ____
____
____
____
____

- **Follow-up Questions:** When would you use urine pregnancy test kits?

Notes: ____
____
____
____
____

Where would you get it from?

Notes: ____
____
____
____
____

What difficulties do you have in getting or using the kits?

Notes: ____
____
____
____
____

What do you think about getting it from a CHV?

Notes: ____
____
____
____
____

What would be the benefits to getting it from a CHV?

Notes: ____
____
____
____
____

What would be the drawbacks to getting it from a CHV?

Additional File 1

Pre-intervention focus group discussion guide for women (English)

Notes: ____
____
____
____
____

- **Probe Question:** How do you know what to do if the test is positive?

Notes: ____
____
____
____
____

- **Follow-up Questions:** Where would you go if you had a positive pregnancy test?

Notes: ____
____
____
____
____

What would you do if you were unhappy about being pregnant?

Notes: ____
____
____
____
____

At what point in pregnancy would you go to a health centre?

Notes: ____
____
____
____
____

What would be the benefits to going for ANC early in your pregnancy?

Notes: ____
____
____
____
____

What do you think about getting counselling and referral from a CHV?

Notes: ____
____
____

____

____

What do you think about getting counselling and referral by private SMS or phone call?

Notes: ____

____

____

____

____

- **Probe Question:** What would you do if the test was negative and you did not want to be pregnant?

Notes: ____

____

____

____

____

- **Follow-up Questions:** Would you seek out family planning?

Notes: ____

____

____

____

____

Where would you go for family planning?

Notes: ____

____

____

____

____

What difficulties would you face in getting family planning?

Notes: ____

____

____

____

____

What do you think about getting FP counselling and referral to FP clinic from a CHV?

Notes: ____

____

____

___

What do you think about getting counselling and referral by private SMS or phone call?

Notes: ___

- **Exit Questions:** Is there anything else you want us to know about receiving urine pregnancy test kits from CHVs?

Notes: ___

Is there anything else you want us to know about receiving counselling and referral from CHVs?

Notes: ___

Is there anything else you want us to know about receiving counselling and referral from private SMS or phone call?

Notes: ___

Additional File 1

Pre-intervention focus group discussion guide for men (English)

Pre Intervention Focus Group Guide – Men

- **Probe Question:** How do you know when your wife is pregnant?

Notes: ____
____
____
____
____

- **Follow-up question:** Do you know what a urine pregnancy test kit is?

Notes: ____
____
____
____
____

Does your wife use the urine pregnancy test kit?

Notes: ____
____
____
____
____

(If Yes) When would she use a urine pregnancy test kit?

What is the reason why she would use it?

Notes: ____
____
____
____
____

(If No) What is the reason why she wouldn't use?

Notes: ____
____
____
____
____

What is your perception about the use of Urine pregnancy test kit?

Notes: ____
____
____
____
____

Additional File 1

Pre-intervention focus group discussion guide for men (English)

What is the perception of men in this community about a CHV?

Notes: ____

What is the perception of men in this community about a CHV offering services to their women?

Notes: ____

What is the perception of men in this community regarding CHVs /healthcare workers on the issue of confidentiality when offering services to their wives and members of the community?

Notes: ____

What benefits do you receive from the CHVs?

Notes: ____

What difficulties do you have with CHVs?

Notes: ____

What would be the benefits of getting the kit from a CHV?

Notes: ____

What would be the drawbacks of getting it from a CHV?

Pre-intervention focus group discussion guide for men (English)

Notes: ____
____
____
____
____

Would you ever consider purchasing a kit for your wife?

Notes: ____
____
____
____
____

Where would you purchase (or get) a urine pregnancy testing kit?

Notes: ____
____
____
____
____

Would your religious beliefs affect your feelings about pregnancy testing?

(If Yes): How, Or in what way?. And for family planning?

Notes: ____
____
____
____
____

Would your traditional and cultural beliefs and perceptions affect your feelings about pregnancy testing?

(If Yes): How, Or in what way? And for family planning?

Notes: ____
____
____
____
____

- **Probe Question:** What would you do if she tests positive? Or how would you know what to do if her pregnancy test is positive?

Notes: ____
____
____
____
____

Additional File 1

Pre-intervention focus group discussion guide for men (English)

- **Follow-up Questions:** Where would you take her if the pregnancy test is positive?

Notes: ____

Where would you recommend her to go if her pregnancy test is positive?

Notes: ____

What would you do as a couple if you were unhappy about the pregnancy?

Notes: ____

Where would go? Or who would you go to?

(If Yes): Give reason.

(If No): Give reason.

Notes: ____

Where would you go for the ANC services?

Notes: ____

What would be the unseen difficulties in getting the ANC services?

Notes: ____

Additional File 1

Pre-intervention focus group discussion guide for men (English)

What are the benefits of getting ANC services?

Notes: ____
____
____
____
____

What are some of the difficulties women face in pregnancy?

Notes: ____
____
____
____
____

What if the pregnancy test is negative…and you don't want another baby? Where would you go or what would you do? Or you do not want another baby?

Notes: ____
____
____
____
____

- **Probe Question:** What is your perception about Family planning and its use?

Notes: ____
____
____
____
____

- **Follow-up Question:** What are the benefits of using FP?

Notes: ____
____
____
____
____

What are the drawbacks or risks of using FP?

Notes: ____
____
____
____
____

What are your feelings about your wife using family planning?

Additional File 1

Pre-intervention focus group discussion guide for men (English)

Notes: ____

What do the men in your community think about specific FP methods? (implants, IUCDs, Depo, condoms, BTL)

Notes: ____

- **Probe Question:** Would you accompany your wife to the clinic?

Notes: ____

- **Follow-up Question:** What would be the benefits?

Notes: ____

What would be the drawbacks?

Notes: ____

- **Probe Question:** What do they think about CHV counseling?

Notes: ____

- **Follow-up Question:** What do they think about phone-based counseling?

Notes: ____

____

____

Is there any other thing you want us to know about UPT, its use and CHV providing it to your wives?

Notes: ____

____

____

____

____

- **Exit question:** Explain the intervention we are planning (CUPTs)-ask what their opinions of this program?

Notes: ____

____

____

____

____

What are some benefits of this program?

Notes: ____

____

____

____

____

What are some risks/drawbacks?

Notes: ____

____

____

____

____

What should we exempt from this program?

Notes: ____

____

____

____

____

What are your recommendations for the program in future?

Notes: ____

____

____

____

____

Additional File 1

Post-intervention focus group discussion guide for Community Health Volunteers (English)

Post Intervention Focus Group Guide – Community Health Volunteers

- **Probe Question:** How was your experience in providing urine pregnancy test kits?

Notes: ____

____

____

____

____

- **Follow-up Questions**: How comfortable did you feel providing urine pregnancy kits?

Notes: ____

____

____

____

____

What are some examples of when you felt uncomfortable (*or did not feel comfortable)* providing urine pregnancy kits?

Notes: ____

____

____

____

____

Why do you think there were no participants who reported being raped?

Notes: ____

____

____

____

____

What benefits did you experience in providing urine pregnant kits?

Notes: ____

____

____

____

____

What benefits did the women experience?

Notes: ____

____

____

Additional File 1

Post-intervention focus group discussion guide for Community Health Volunteers (English)

What difficulties did you face in providing urine pregnancy kits?

Notes:

What difficulties did the women face?

Notes:

Would you want to continue providing urine pregnancy test kits? Why or why not?

Notes:

- **Probe Question**: How was your experience in counseling & referring women about what to do if they have a positive pregnant test?

Notes:

- **Follow-up Questions:** How comfortable did you feel about counseling & referring women about what to do if they have a positive pregnancy test?

Notes:

How comfortable did you feel counseling & referring women who were unhappy about being pregnant?

Notes:

Additional File 1

Post-intervention focus group discussion guide for Community Health Volunteers (English)

How was your experience counseling & referring women who said they wanted an abortion?

Notes: ____

____

____

____

____

What benefits did you experience in counseling & referring women with a positive pregnancy test?

Notes: ____

____

____

____

____

What benefits did the women experience?

Notes: ____

____

____

____

____

What difficulties did you face in providing counseling & referral for women with a positive pregnancy test?

Notes: ____

____

____

____

____

What difficulties did the women face?

Notes: ____

____

____

____

____

Would you want to continue providing counseling & referral for women with a positive pregnant test?

Notes: ____

____

____

____

____

- **Probe Question**: How was your experience in counseling & referring women about what to do if they have a negative pregnant test?

Additional File 1

Post-intervention focus group discussion guide for Community Health Volunteers (English)

Notes: ____
____
____
____
____

- ***Follow-up Questions:*** How comfortable did you feel about counseling & referring women about what to do if they have a negative pregnant test?

Notes: ____
____
____
____
____

How comfortable did you feel counseling & referring women who did not want to be pregnant?

Notes: ____
____
____
____
____

How comfortable did you feel counseling & referring women wanted to be pregnant?

Notes: ____
____
____
____
____

What benefits did you experience in counseling & referring women with a negative pregnancy test?

Notes: ____
____
____
____
____

What benefits did the women experience?

Notes: ____
____
____
____
____

What difficulties did you face in providing counseling & referral for women with a negative pregnancy test?

Additional File 1

Post-intervention focus group discussion guide for Community Health Volunteers (English)

Notes: ____
____
____
____
____

What difficulties did the women face?

Notes: ____
____
____
____
____

Would you want to continue providing counseling & referral for women with a negative pregnant test?

Notes: ____
____
____
____
____

- **Probe Question:** Was there ever a time when you did not provide post-test counseling and referral?

Notes: ____
____
____
____
____

- **Follow-up questions:** What are some examples of when & why you did not provide post-test counselling?

Notes: ____
____
____
____
____

How did you give women the option of phone-based counseling?

Notes: ____
____
____
____
____

Why do you think participants chose phone-based counselling?

Notes: ____
____
____

Why do you think women who chose phone-based counseling never contacted the RA? Did you provide them with the cards to contact the RA?

Notes: ____________________

Did you feel they needed CHV-based counselling in addition to their desired phone-based counselling?

Notes: ____________________

- ***Exit Questions:*** Is there anything else you want us to know about how to change or improve the process for providing urine pregnancy test kits in the future?

Notes: ____________________

Is there anything else you want us to know about how to change or improve the process providing counseling and referral for women who are pregnant in the future?

Notes: ____________________

Is there anything else you want us to know about how to change or improve the process providing counseling and referral for women who are not pregnant in the future?

Notes: ____________________

Additional File 1

Post-intervention focus group discussion guide for Community Health Volunteers (English)
